# Neonatal exposure of 17β-estradiol has no effects on mutagenicity of 7,12-dimethylbenz [a] anthracene in reproductive tissues of adult mice

**DOI:** 10.1186/s41021-015-0011-y

**Published:** 2015-07-30

**Authors:** Zhuhong Zhang, Haifang Li, Mugimane G. Manjanatha, Tao Chen, Nan Mei

**Affiliations:** Division of Genetic and Molecular Toxicology, National Center for Toxicological Research, Jefferson, AR 72079 USA; Tianjin Medical University General Hospital, Tianjin, 300052 China; Xinjiang Institute for Food and Drug Control, Urumqi, Xinjiang 830004 China

**Keywords:** 17 β-estradiol, Neonatal exposure, Mammary gland, Ovary, Mutagenicity

## Abstract

**Introduction:**

Biological studies in animals and epidemiological findings in humans clearly demonstrate that estrogens including 17β-estradiol (E2) are weak carcinogens via both genetic and epigenetic mechanisms. Carcinogenesis analyses have indicated that female mice exposed to E2 as neonates develop more mammary and ovarian tumors when compared to adult exposures. In the present study, Big Blue transgenic mice were used to investigate the effects of E2 on mutagenicity of 7,12-dimethylbenz [a] anthracene (DMBA), a genotoxic carcinogen, in mammary gland and ovary following neonatal exposure.

**Results:**

DMBA treatment resulted in significant increases in *cII* mutant frequencies (MFs) in both mammary glands and ovaries, with A:T → T:A transversion as the predominant type of mutation. However, co-exposure to E2 daily for the first 5 days after birth and to DMBA at 6 months of age did not significantly increase *cII* MFs compared to DMBA treatment alone. Further, there were also no significant differences in mutational spectra between DMBA exposure alone and E2 + DMBA treatment.

**Conclusion:**

These results suggest that early life exposures of mice to estrogens like E2 do not enhance mutagenicity by subsequent exposure to a chemical like DMBA in later life.

**Electronic supplementary material:**

The online version of this article (doi:10.1186/s41021-015-0011-y) contains supplementary material, which is available to authorized users.

## Introduction

Estrogens are mainly known for their roles in female reproductive tissues, but also have a vast array of functions in other tissues, including adipose tissue, bone, brain, skeletal muscle, skin, and the vasculature [1]. Human exposure to low doses of endogenous estrogens, estrogenic drugs, phytoestrogens, and xenoestrogens has the potential to improve health or disrupt normal endocrine activity [2]. 17β-estradiol (E2), as the primary endogenous estrogen produced in the ovary in premenopausal women, profoundly effects sexual differentiation, reproductive function, and behavior; in addition to other activities, such as regulating lipid profiles and the levels of blood clotting factors. E2 has been used for hormone replacement therapy in postmenopausal women, as it can remit menopause symptoms and improve the quality of life for many women [3]. However, numerous studies have suggested that there are increased risks for breast and ovarian cancers associated with hormone replacement therapy [4, 5].

Animal studies and epidemiological findings in humans clearly demonstrate that estrogens, including E2, are weak carcinogens via both genetic and epigenetic mechanisms [6]. It has been reported that E2 induces DNA damage and causes a relatively low frequency of gene mutations, and the genotoxic activity of E2 is most likely induced by catecholestrogen metabolites after metabolic activation [7]. The effects of estrogens appear to be tissue-specific, as tumor induction is primarily in the uterus, mammary glands, and ovaries. Carcinogenesis studies have indicated that female mice exposed to E2 as neonates developed more mammary and ovarian tumors when compared to adult exposure [8, 9]. In addition, E2 promotes the development of hepatic neoplasms in rats [10, 11].

It has been reported that exposure to chemicals (i.e., endocrine disrupting chemicals interfering with the body’s endocrine system) during early stages of development can disrupt normal patterns of development and alter disease susceptibility later in life [12]. About three decades ago, a concern was expressed about the levels of phytoestrogens in soy infant-formula presenting the risk of adverse effects. High circulating levels of genistein have been determined in infants fed soy-based formulas [13]. It has been reported that neonatal exposure to genistein disrupts the female mouse reproductive tract and subsequently contributes to infertility [14]. However, there is limited information regarding long-term effects of infant exposure to soy-based infant formulas, and the effect of early life exposures to phytoestrogens on animal susceptibility to subsequent chemical insults in later life [15].

Previously, we evaluated 7,12-dimethylbenz [a] anthracene (DMBA)-induced mutagenicity in the heart, mammary gland, and uterine tissues as well as DMBA-induced carcinogenicity in the mammary and uterine dysplasia in both ovariectomized (OVX) and ovary intact (INT), 7 week-old Big Blue transgenic rats fed E2 and soy isoflavones for up to 20 weeks [16–18]. Although feeding isoflavones or E2 did not cause any significant changes in the DMBA-induced mutagenicity in the mammary or uterus of both OVX and INT rats, there were significant increases in the incidence of ductal hyperplacia and adenoma/adenocarcinoma in the mammary gland of INT Big Blue rats and dysplasia in the uterus of OVX rats fed E2 or isoflavones with or without DMBA. However, unlike the reproductive tissues, where E2 was not beneficial, the DMBA-induced mutagenicity in the heart was significantly modulated by E2 suggesting a beneficial effect [17]. In this study, using E2 as a model estrogen, we investigated whether or not neonatal exposure to E2 modifies the mutant frequency (MF) in Big Blue mice treated at later life with DMBA, a mammary gland and ovary mutagen and carcinogen.

## Materials and methods

### Chemicals and reagents

DMBA and E2 were purchased from Sigma (St. Louis, MO). The RecoverEase DNA Isolation Kit, Transpack packaging extract, and the Escherichia coli G1250 strain were obtained from Agilent Technologies (Santa Clara, CA). PCR Master Mix was purchased from Promega (Madison, WI), and CEQ Dye Terminator Cycle Sequencing kits were obtained from Beckman Coulter (Fullerton, CA).

### Animals and treatments

During the course of animal experiment, we followed the recommendations set forth by our Institutional Animal Care and Use Committee for the handling, maintenance, treatment, and sacrifice of the animals. Six pregnant female Big Blue C57BL/6 transgenic mice (homozygous for the transgene) were obtained from Taconic Farms (Germantown, NY). These mice were bred with non-transgenic C3H male mice by the supplier. Twenty female B6C3F1 offspring (heterozygous for the transgene) were pooled and randomly divided into 4 groups at birth (i.e., 5 mice in each group). Six to eight pups were assigned to each foster mother [19]. Pups of two groups (i.e., 10 pups) were given 10 mg E2 per kg body weight by subcutaneous injection on each of the first 5 days of life and two groups were not treated. After weaning, the mice were fed NIH-31IR diet (Purina Mills, Brentwood, MO). At 6 months of age, the mice in one group each with or without neonatal exposure to E2 were treated with 20 mg DMBA per kg body weight by gavage twice within a week. Our dose choice of E2 or DMBA was based on previous studies where DMBA alone resulted in significant increases in gene mutant frequencies [20, 21] while E2 alone aderministered for up to 2 years induced tumors in various organs of mice [22], and combined treatment with E2 and DMBA induced more ovarian tumors [8]. Six weeks after DMBA treatment, the mice in the four groups were sacrificed, and the mammary glands and ovaries were isolated, frozen quickly in liquid nitrogen, and stored at −80 °C for later assays of *cII* mutations.

### cII mutant assay

High molecular-weight genomic DNA was extracted from ovaries and mammary glands using the RecoverEase DNA Isolation Kit [23] and the *cII* mutagenicity assay was performed using the method previously described [24].

### Sequence analysis of cII mutants

The *cII* mutant plaques from control and treated mice were isolated and sequenced as previously reported [25].

### Statistical analyses

Analyses were performed using SigmaStat 11.0 (Systst Software Inc., San Jose, CA). Data are expressed as the mean ± standard error of mean (SEM) from 3 or 5 mice per group. Statistical significance was determined by one-way analysis of variance (ANOVA) followed by the Holm-Sidak test for comparison of multiple treatment groups. Mutation spectra were compared using the computer program written by Cariello et al. [26] for the Monte Carlo analysis developed by Adams and Skopek [27]. 

## Results

### Mutant frequency

**Fig. 1:**
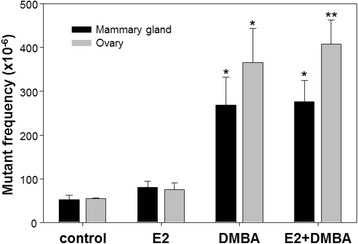
Mutant frequencies in the *cII* gene in mammary gland and ovary of mice. Big Blue transgenic mice were exposed to E2 during the first 5 days of life and/or treated with DMBA at 6 months of age. Significant differences were found between DMBA and control groups, between E2 + DMBA and control groups, between DMBA and E2-only groups, and between E2 + DMBA and E2-only groups, respectively, for the both tissues (* *p* < 0.05 and ** *p* < 0.01). The data represent the mean ± S.E.M. from 3 ~ 5 mice in each group

MFs were determined for the mammary and ovary *cII* gene of the Big Blue mice treated with DMBA at 6 months with or without neonatal exposure to E2 (Fig. 1). For the mammary gland, the MF of the control group was 52.6 ± 9.8 (SEM) × 10^−6^. The MF in the E2 group was 79.9 ± 15.2 × 10^−6^, which was slightly higher than the unexposed control group but not significantly different (ANOVA, Holm-Sidak test). The MFs in the DMBA and E2 + DMBA groups were 268.9 ± 63.1 × 10^−6^ and 276.6 ± 48.6 × 10^−6^, respectively, which were significantly higher than that of the unexposed control group and the E2 alone group (*p* < 0.05). In the ovary, the MFs of the DMBA and E2 + DMBA groups were 366.2 ± 78.1 × 10^−6^ and 408.7 ± 54.3 × 10^−6^, respectively; both significantly higher than that of the unexposed control (54.3 ± 1.2 × 10^−6^) and E2 only group (75.8 ± 15.0 × 10^−6^). There was no significant difference between the E2-only group and the control group. These results indicate that DMBA induced significant increases in *cII* MF both in the mammary gland and ovary and that the neonatal exposure to E2 did not affect the mutation induction by DMBA in the two tissues.

### Mutation analysis

We examined the types of mutations induced by DMBA with or without neonatal exposure to E2 in mammary glands and ovary tissues. The *cII* gene mutations in the mammary gland were analyzed by DNA sequencing (Additional file 1: Table S1); and 33 independent mutations were identified from the control group, 40 from the E2-only group, 66 from the DMBA group, and 91 from the E2 + DMBA group (Table 1). A pairwise multiple comparisons of these mutational spectra were performed [28]. There were significant differences in the type of mutations between the DMBA and control groups. Also, the mutations in the E2 + DMBA group and the E2-only group differed significantly (*p* < 0.001). However, there were no significant differences between the E2-only and unexposed control groups (*p* = 0.90), and between the DMBA and E2 + DMBA groups (*p* = 0.99). Among the mutations, the major type of mutation was G:C → A:T transition in the unexposed control (43 %) and E2 treated (45 %) groups, whereas a A:T → T:A transversion was the predominant mutation in the DMBA (50 %) and E2 + DMBA (53 %) groups (Table 1). We also conducted DNA sequence analysis of the ovary *cII* gene (Additional file 1: Table S2). We identified 38 independent mutations from the control group, 25 from the E2-only group, 98 from the DMBA group, and 114 from the E2 + DMBA group (Table 1). Consistent with that of mammary tissue, there was a significant difference in the types of mutations between E2 + DMBA group and E2-only group (*p* < 0.01). The types of mutations in the DMBA group and control group also differed significantly (*p* = 0.004). However, there were no significant differences between the E2 and unexposed control groups (*p* = 0.92), and between the DMBA and E2 + DMBA groups (*p* = 0.62). The most common type of mutations induced by DMBA in the ovary was also A:T → T:A transversion, which accounted for 38 % and 33 % of mutations in the DMBA and E2 + DMBA groups, respectively. Whereas G:C → A:T transition was the major type of mutation in the unexposed control (42 %) group and E2-only (44 %) group, respectively (Table 1).

**Table 1:** Summary of independent *cII* mutations in mammary gland and ovary of Big Blue transgenic mice exposed to E2 at the first 5 days of life and/or treated with DMBA at 6 months of age

Tissue	Type of mutation	Control		E2^*a*^		DMBA^*b*^		E2 + DMBA^*c, d*^	
		Number	%	Number	%	Number	%	Number	%
Mammary gland	G:C → C:G	3	9	4	10	4	6	4	4
	G:C → A:T	14	43	18	45	5	8	8	9
	G:C → T:A	7	21	6	15	13	20	14	16
	A:T → T:A	1	3	4	10	33	50	48	53
	A:T → C:G	3	9	3	8	3	4	4	4
	A:T → G:C	2	6	1	2	5	8	9	10
	Frameshift	3	9	4	10	3	4	3	3
	Tandem	0	0	0	0	0	0	1	1
	Total mutants screened	33	100	40	100	66	100	91	100
Ovary	G:C → C:G	2	5	2	8	11	11	13	11
	G:C → A:T	16	42	11	44	12	12	19	17
	G:C → T:A	9	24	6	24	22	23	20	17
	A:T → T:A	3	8	1	4	37	38	37	33
	A:T → C:G	1	3	1	4	5	5	6	5
	A:T → G:C	2	5	0	0	9	9	10	9
	Frameshift	5	13	4	16	2	2	7	6
	Tandem	0	0	0	0	0	0	2	2
	Total mutants screened	38	100	25	100	98	100	114	100

^*a*^No significant difference from control group

^*b*^Spectrum from DMBA-treated mice was significantly different from control and E2-only groups (*p* < 0.0001)

^*c*^Spectrum from the mice exposed to E2 and DMBA was significantly different from control and E2-only groups (*p* < 0.01 in ovary and *p* < 0.0001 in mammary gland)

^*d*^No significant difference from DMBA group

## Discussion

Breast cancer is the most common cancer (25.1 %) in women globally, while ovarian cancer is the 7th most common cancer (3.6 %) [29]. Since 2000, breast cancer incidence in the U.S. has decreased, partially due to the reduced use of hormone replacement therapy. However, it remains the leading cause of cancer deaths in American women with about 40,000 deaths each year [30]. In 2014, there were 22,000 new cases of ovarian cancer in the U.S., with about 14,270 deaths in 2014 [31]. Among various factors related to reproductive cancers, endogenous estrogens and exogenous hormones are the key risk factors.

Chemicals with estrogenic activity are derived from thousands of natural and synthetic processes; therefore, human exposure to estrogenic chemicals is ubiquitous [2]. Soy infant formula containing phytoestrogens has been fed to infants for ~60 years as a supplement to or replacement for human milk or cows’ milk. Recently, the concerns for the reproductive and developmental toxicity of soy infant formula have been raised, and one of numerous data gaps is that early life exposure to phytoestrogens may alter risk for hormonally dependent diseases, such as breast cancer [15]. In addition, it has been suggested that prenatal or neonatal exposure to endocrine-disrupting compounds can alter the hormonal milieu, reproductive tissue development, and susceptibility to potential carcinogen exposure in adults [32].

DMBA, a polycyclic aromatic hydrocarbon and a model carcinogen, has been extensively used in experimental carcinogenesis studies. Previously, we demonstrated that genistein, an alternative to hormone replacement therapy in postmenopausal women, showed no effects on DMBA**-**induced mutagenicity in livers [11], mammary glands [18], and uterus in Big Blue transgenic rats [16]. In this study, Big Blue transgenic mice were injected subcutaneously with E2 on the first 5 days of their life and then received DMBA at age of 6 months. For both mammary gland and ovary tissues, there were no significant differences in MFs in the *cII* gene between the control and E2-only groups, and between the two DMBA groups with or without neonatal exposure to E2; however, the DMBA treatment groups showed significantly higher MFs than the control and E2 alone groups (Fig. 1). Similarly, we also observed similar patterns of mutation spectra, with A:T → T:A transversions as the predominant mutation in the DMBA-treated groups compared to G:C → A:T transitions as the main mutation in the control and E2-only groups (Table 1). These results indicate that DMBA induced significant *cII* MFs both in the mammary gland and ovary tissues; however, neonatal exposure to E2 did not significantly affect the mutagenicity induced by DMBA exposure in later life both in the mammary gland and ovary of Big Blue mice. These results indicate that under the experimental conditions used in this study, elevated levels of estrogen in neonates do not promote chemical-induced mutations in adults.

When female C3/129 mice were given E2 injections for the first 5 days after birth and gastric intubations of DMBA at an age of 70 days, more ovarian tumors (78 %) of the granulose cell type were observed at the age of 1 year compared to those in DMBA alone group (33 %) [8]. In addition, about 59 % of mice developed tumors at 20 weeks of age, whereas no mice had ovarian tumor in the DMBA-alone group. Similar results also were observed in the incidence of mammary dysplasia in BALB/c mice [9]. Recently, soy diet has been found to promote granulose cell tumors in C57BL/6 J/129S7/Sv mixed mice [33]. Although there is genetic variation in physiological sensitivity to E2 between strains of mice, C57BL/6 is one of mouse strains that are highly responsive to E2 [34, 35]. Our results for the *cII* MFs in female B6C3F1 mice (C57BL/6 as one of the parental strains) were not directly correlated with the incidences of breast or ovarian tumors, suggesting that induction of tumors by early exposure to estrogens is not via increasing mutagenicity; thus other factors associated with the initiation, promotion, and progression of cancers may be involved. Although many aspects are still unknown, estrogen receptor-mediated effects on cell proliferation contribute to the weight of evidence for estrogen-mediated cancer [36]. It has been reported that genistein modulates estrogen-receptor expression and signaling in COV434 cells derived from a primary human juvenile granulose cell tumor, promoting cell growth [33]. In addition, the nature of genetic differences in mouse strains and subspecies is a major source of variation in susceptibility to endocrine disruption by estrogens [37].

## Conclusion

 In 2008, the National Toxicology Program (NTP) reported that there was some evidence (or equivocal evidence) of carcinogenic activity of genistein in female rats, with acceleration of the onset of aberrant estrous cycles when rats were continuously exposed to genistein from conception through weaning (21-day-old), 20 weeks, and 2 years, respectively, and all rats were sacrificed after 2-year study [38]. Regarding soy infant formula containing phytoestrogens, there was insufficient data on reproductive toxicity in humans and animals [39] and recently, a NTP expert panel has summarized critical data gaps and research needs on pharmacokinetics, human epidemiology, and experimental animals [15]. The results in this study demonstrates that neonatal exposure to E2 did not affect the mutagenicity in mammary gland and ovary induced by DMBA in mice, and provides a piece of information concerning the effect of early life exposures to estrogens on the mutagenicity or carcinogenicity of subsequent chemical insults in later life.

## Additional file

Additional file 1:
**Supplementary Tables 1–2.**
