# Supplementary material for: Neonatal exposure of 17β-estradiol has no effects on mutagenicity of 7,12-dimethylbenz [a] anthracene in reproductive tissues of adult mice
Source: Genes Environ. 2015 Jul 30;37:16. doi: 10.1186/s41021-015-0011-y (PMC4918036; doi:10.1186/s41021-015-0011-y)
Supplement: Additional file 1: — Supplementary Tables 1–2. [file 41021_2015_11_MOESM1_ESM.doc]

**Supplementary Table 1.** Mutations in the *cII* gene of mammary glands of Big Blue transgenic mice exposed to E2 at the first 5 days of life and/or treated with DMBA at 6 months of age.

| Position^a^ | Mutation^b^ | Amino acid change | Sequence context  5’ → 3’^c^ | Number of mutations (independent) | | | |
| --- | --- | --- | --- | --- | --- | --- | --- |
|  |  |  |  | Control | E2 | DMBA | E2+DMBA |
| -15 | A → T | N/A | ctaAGGaaa | 1 |  |  |  |
| -14 | G → A | N/A | ctaAGGaaa |  | 1 |  |  |
|  | G → T | N/A | ctaAGGaaa |  |  | 1 |  |
| -3 | C → G | N/A | ttaCATatg |  |  | 1 |  |
| 1 | A → T | Met → Leu | catATGgtt |  |  |  | 2 (1) |
| 2 | T → C | Met → Thr | catATGgtt |  |  | 1 | 1 |
| 3 | G → A | Met → Ile | catATGgtt |  |  |  | 1 |
| 4 | G → C | Val → Leu | atgGTTcgt |  | 1 |  |  |
| 5 | T → A | Val → Asp | atgGTTcgt |  |  |  | 1 |
| 13 | A → T | Asn → Tyr | gcaAACaaa |  |  | 1 |  |
| 16 | A → T | Lys → Stop | aacAAAcgc |  |  | 1 | 1 |
| 25 | G → A | Glu → Lys | aacGAGgct |  | 1 |  |  |
|  | G → T | Glu → Stop | aacGAGgct |  | 1 | 1 |  |
| 26 | A → C | Glu → Ala | aacGAGgct | 1 |  |  |  |
|  | A → G | Glu → Gly | aacGAGgct | 1 |  |  | 1 |
| 28 | G → A | Ala → Thr | gagGCTcta | 1 |  |  |  |
| 29 | C → T | Ala → Val | gagGCTcta | 1 |  |  |  |
| 34 | C → T | Arg → Stop | ctaCGAatc |  | 1 |  | 1 |
|  | C → G | Arg → Gly | ctaCGAatc |  |  | 1 |  |
| 35 | G → A | Arg → Gln | ctaCGAatc |  | 1 |  |  |
| 37 | A → T | Ile → Phe | cgaATCgag |  |  | 1 | 2 (2) |
| 40 | G → A | Glu → Lys | atcGAGagt | 1 | 1 |  | 2 (1) |
| 41 | A → G | Glu → Gly | atcGAGagt |  |  | 1 |  |
|  | A → T | Glu → Val | atcGAGagt |  |  | 1 | 2 (2) |
| 46 | G → C | Ala → Pro | agtGCGttg |  |  |  | 1 |
| 49 | T → A | Leu → Met | gcgTTGctt |  |  | 1 |  |
| 50 | T → A | Leu → Stop | gcgTTGctt |  |  | 1 |  |
| 51 | G → C | Leu → Phe | gcgTTGctt |  |  |  | 1 |
|  | G → T | Leu → Phe | gcgTTGctt |  |  | 2 (1) |  |
| 52 | C → G | Leu → Val | ttgCTTaac |  | 1 |  |  |
| 53 | T → A | Leu → His | ttgCTTaac |  |  | 2 (2) | 2 (2) |
| 56 | A → T | Asn → Ile | cttAACaaa |  |  | 2 (2) |  |
| 58 | A → T | Lys → Stop | aacAAAatc |  |  | 2 (2) |  |
| 59 | A → T | Lys → Ile | aacAAAatc |  |  |  | 1 |
| 62 | T → G | Ile → Ser | gcaATCgca |  |  | 1 |  |
| 64 | G → A | Ala → Thr | atcGCAatg | 5 (3) | 1 | 1 |  |
| 68 | T → A | Met → Lys | gcaATGctt |  |  | 1 |  |
| 71 | T → A | Leu → His | atgCTTgga |  |  |  | 3 (2) |
|  | T → C | Leu → Pro | atgCTTgga |  |  |  | 2 (2) |
| 74 | G → C | Gly → Ala | cttGGAact |  |  |  | 1 |
| 79 | G → T | Glu → Stop | actGAGaag | 1 |  |  |  |
| 83 | A → T | Lys → Met | gagAAGaca |  |  | 1 | 2 (2) |
| 86 | C → A | Thr → Lys | aagACAgcg | 1 |  | 1 |  |
| 89 | C → A | Ala → Glu | acaGCGgaa | 1 |  |  |  |
|  | C → T | Ala → Val | acaGCGgaa | 4 (2) | 1 | 1 |  |
| 91 | G → T | Glu → Stop | gcgGAAgct |  |  |  | 1 |
| 92 | A → G | Glu → Gly | gcgGAAgct |  |  | 1 |  |
| 94 | G → A | Ala → Thr | gaaGCTgtg |  | 1 |  |  |
| 98 | T → A | Val → Glu | gctGTGggc |  |  | 3 (2) | 7 (3) |
|  | T → C | Val → Ala | gctGTGggc |  | 1 |  |  |
|  | T → G | Val → Gly | gctGTGggc |  |  |  | 1 |
| 100 | G → T | Gly → Cys | gtgGGCgtt |  |  | 2 (1) | 2 (2) |
| 103 | G → A | Val → Ile | ggcGTTgat |  |  | 1 |  |
|  | G → T | Val → Phe | ggcGTTgat |  | 1 |  | 1 |
| 104 | T → A | Val → Asp | ggcGTTgat |  |  | 1 | 2 (2) |
|  | T → C | Val → Ala | ggcGTTgat |  |  |  | 1 |
| 106 | G → T | Asp → Tyr | gttGATaag |  |  | 1 | 2 (2) |
| 106-107 | + G | Frameshift | gttGATaag |  |  | 1 |  |
| 107 | A → T | Asp → Val | gttGATaag |  | 1 |  | 1 |
|  | A → C | Asp → Ala | gttGATaag |  | 1 |  |  |
| 108 | T → A | Asp → Glu | gttGATaag |  |  | 1 |  |
| 110 | A → T | Lys → Met | gatAAGtcg |  |  | 2 (2) |  |
| 112 | T → A | Ser → Thr | aagTCGcag |  |  | 1 | 1 |
|  | T → C | Ser → Pro | aagTCGcag | 1 |  | 1 |  |
| 113 | C → T | Ser → Leu | aagTCGcag | 1 | 1 |  |  |
|  | C → G | Ser → Trp | aagTCGcag |  | 1 |  |  |
| 116 | A → T | Gln → Leu | tcgCAGatc |  |  |  | 2 (2) |
| 119 | T → A | Ile → Asn | cagATCagc |  |  |  | 1 |
| 121 | A → T | Ser → Cys | atcAGCagg |  |  |  | 1 |
| 124 | A → T | Arg → Trp | agcAGGtgg |  |  |  | 2 (2) |
| 127 | T → C | Trp → Arg | aggTGGaag |  |  |  | 1 |
| 127-128 | + T | Frameshift | aggTGGaag |  |  |  | 1 |
| 133 | A → T | Arg → Trp | aagAGGgac |  | 1 | 2 (2) | 1 |
| 139 | T → A | Trp → Arg | gacTGGatt |  |  | 1 | 2 (2) |
| 141 | G → A | Trp → Stop | gacTGGatt | 1 |  |  |  |
| 145 | C → A | Pro → Thr | attCCAaag |  | 1 |  | 1 |
| 146 | C → A | Pro → Gln | attCCAaag | 1 |  | 1 |  |
| 150 | G → T | Lys → Asn | ccaAAGttc |  | 1 | 1 |  |
| 152 | T → G | Phe → Cys | aagTTCtca |  |  | 1 |  |
| 154 | T → A | Ser → Thr | ttcTCAatg |  |  | 1 | 1 |
| 155 | C → A | Ser → Stop | ttcTCAatg |  |  |  | 1 |
| 161 | T → A | Leu → Gln | atgCTGctt |  |  | 1 | 4 (3) |
| 163 | C → G | Leu → Val | ctgCTTgct | 1 |  |  |  |
| 164 | T → A | Leu → His | ctgCTTgct |  |  |  | 2 (2) |
|  | T → C | Leu → Pro | ctgCTTgct |  |  |  | 2 (1) |
| 166 | G → A | Ala → Thr | cttGCTgtt |  |  | 1 |  |
|  | G → T | Ala → Ser | cttGCTgtt |  |  | 1 |  |
| 169 | G → T | Val → Phe | gctGTTctt |  |  | 1 |  |
| 172-220 | deletion | Frameshift |  |  |  | 1 |  |
| 173 | T → A | Leu → His | gttCTTgaa |  |  |  | 1 |
| 175 | G → T | Glu → Stop | cttGAAtgg |  |  | 1 |  |
| 178/185 | + G | Frameshift | gaaTGGGGGGTCgtt | 1 | 2 (2) |  |  |
| 178 | T → A | Trp → Arg | gaaTGGggg |  | 1 | 2 (2) | 1 |
| 179 | G → T | Trp → Leu | gaaTGGggg |  | 1 |  |  |
| 179-184 | − G | Frameshift | gaaTGGGGGGTCgtt | 1 | 4 (2) | 1 | 1 |
| 181 | G → T | Gly → Trp | tggGGGgtc | 1 |  |  | 1 |
| 182 | G → T | Gly → Val | tggGGGgtc |  |  | 1 |  |
| 185 | T → G | Val → Gly | gggGTCgtt | 1 | 1 |  |  |
| 187 | G → T | Val → Phe | gtcGTTgac |  |  |  | 1 |
| 190 | G → T | Asp → Tyr | gttGACgac |  |  |  | 1 |
| 191 | A → T | Asp → Val | gttGACgac | 1 |  | 2 (2) | 1 |
| 193 | G → A | Asp → Asn | gacGACgac |  | 3 (2) |  | 2 (1) |
|  | G → T | Asp → Tyr | gacGACgac | 1 |  |  |  |
| 194 | A → G | Asp → Gly | gacGACgac |  |  | 1 |  |
| 196 | G → A | Asp → Asn | gacGACatg |  | 1 | 1 |  |
|  | G → T | Asp → Tyr | gacGACatg |  | 1 | 1 |  |
| 197 | A → G | Asp → Gly | gacGACatg |  |  |  | 1 |
| 199 | A → T | Met → Leu | gacATGgct |  |  |  | 1 |
| 200 | T → A | Met → Lys | gacATGgct |  |  |  | 3 (3) |
|  | T → G | Met → Arg | gacATGgct |  | 1 |  |  |
| 203 | C → G | Ala → Gly | atgGCTcga | 1 |  |  |  |
| 206 | G → A | Arg → Gln | gctCGAttg |  |  |  | 1 |
| 209 | T → G | Leu → Trp | cgaTTGgcg | 1 |  |  |  |
| 209-210 | TG → AT | Leu → Tyr | cgaTTGgcg |  |  |  | 1 |
| 210 | G → T | Leu → Phe | cgaTTGgcg |  |  |  | 1 |
| 212 | C → G | Ala → Gly | ttgGCGcga |  |  |  | 1 |
|  | C → T | Ala → Val | ttgGCGcga | 1 | 3 (3) |  |  |
| 214 | C → T | Arg → STOP | gcgCGAcaa | 3 (3) | 2 (2) |  | 3 (2) |
| 215 | G → C | Arg → Pro | gcgCGAcaa | 1 |  |  |  |
| 217 | C → T | Gln → Stop | cgaCAAgtt |  | 1 |  |  |
| 218 | A → T | Gln → Leu | cgaCAAgtt |  | 1 |  |  |
| 220 | G → C | Val → Leu | caaGTTgct |  |  | 1 |  |
| 223 | G → C | Ala → Pro | gttGCTgcg |  | 1 | 1 |  |
| 228 | G → A | Ala → Ala | gctGCGatt |  |  |  | 1 |
| 230 | T → A | Ile → Asn | gcgATTctc |  |  | 1 |  |
| 233 | T → A | Leu → His | attCTCacc |  |  | 1 | 1 |
|  | T → G | Leu → Arg | attCTCacc |  |  | 1 | 1 |
| 241-246 | − A | Frameshift | aatAAAAAAcgc | 2 (1) |  |  | 1 |
| 292 | T → A | Stop → Arg | ttcTGAggt |  |  | 1 | 2 (2) |
|  | T → G | Stop → Gly | ttcTGAggt |  |  |  | 3 (2) |
| 293 | G → T | Stop → Leu | ttcTGAggt |  |  |  | 2 (2) |
| 294 | A → G | Stop → Trp | ttcTGAggt |  |  |  | 1 |
|  | A → T | Stop → Cys | ttcTGAggt |  |  |  | 2 (2) |
| Total |  |  |  | 38 (33) | 43 (40) | 69 (66) | 103 (91) |

^a^ Position 1 is the first base of the start codon in the *cII* coding sequence.

^b^ Presented in term of sequence change on nontranscribed DNA strand.

^c^ Uppercase indicates target codon and target bases are underlined.

Abbreviations: −, deletion; +, insertion.

**Supplementary Table 2.** Mutations in the *cII* gene of ovaries of Big Blue transgenic mice exposed to E2 at the first 5 days of life and/or treated with DMBA at 6 months of age.

| Position^a^ | Mutation^b^ | Amino acid change | Sequence context  5’ → 3’^c^ | Number of mutations (independent) | | | |
| --- | --- | --- | --- | --- | --- | --- | --- |
|  |  |  |  | Control | E2 | DMBA | E2+DMBA |
| -14 | G → A | N/A | ctaAGGaaa |  |  |  | 1 |
| -4 | A → G | N/A | tacttacat |  |  |  | 1 |
| 1 | A → G | Met → Val | catATGgtt |  |  |  | 1 |
| 2 | T → C | Met → Thr | catATGgtt | 1 |  | 1 | 1 |
| 3 | G → T | Met → Ile | catATGgtt |  |  |  | 1 |
| 12-14 | ­ A | Frameshift | cgtGCAAACaaa |  |  |  | 1 |
| 13 | A → T | Asn → Tyr | gcaAACaaa |  |  | 1 |  |
| 16 | A → T | Lys → Stop | aacAAAcgc |  |  | 3 (1) | 2 (1) |
| 19 | C → T | Arg → Cys | aaaCGCaac |  | 2 (1) |  |  |
| 20 | G → T | Arg → Leu | aaaCGCaac |  |  | 1 |  |
|  | G → C | Arg → Pro | aaaCGCaac |  | 2 |  | 3 |
| 25 | G → A | Glu → Lys | aacGAGgct |  | 1 |  |  |
|  | G → T | Glu → Stop | aacGAGgct |  |  | 2 (1) |  |
| 26 | A → T | Glu → Val | aacGAGgct |  |  |  | 1 |
| 31 | C → G | Leu → Val | gctCTAcga |  |  |  | 1 |
| 34 | C → T | Arg → Stop | ctaCGAatc | 3 | 2 |  | 2 |
| 35 | G → C | Arg → Pro | ctaCGAatc |  |  |  | 1 |
|  | G → A | Arg → Gln | ctaCGAatc | 1 |  |  |  |
|  | G → T | Arg → Leu | ctaCGAatc |  |  | 2 (1) |  |
| 37 | A → T | Ile → Phe | cgaATCgag |  |  |  | 1 |
| 39 | C → G | Ile → Met | cgaATCgag |  |  |  | 1 |
| 40 | G → A | Glu → Lys | atcGAGagt |  | 1 |  |  |
|  | G → T | Glu → Stop | atcGAGagt |  |  | 2 | 2 (1) |
| 41 | A → G | Glu → Gly | atcGAGagt |  |  | 1 |  |
|  | A → T | Glu → Val | atcGAGagt |  |  | 1 | 1 |
| 42 | G → T | Glu → Asp | atcGAGagt |  |  |  | 3 (2) |
| 43 | ­ AGTGC | Frameshift | gagAGTGCGttg |  |  |  | 1 |
| 46 | G → C | Ala → Pro | agtGCGttg |  |  | 1 |  |
| 47 | C → G | Ala → Gly | agtGCGttg | 1 |  |  |  |
| 49 | T → A | Leu → Met | gcgTTGctt |  |  |  | 3 (1) |
| 50 | T → A | Leu → Stop | gcgTTGctt |  |  | 1 |  |
|  | T → C | Leu → Ser | gcgTTGctt |  |  | 1 |  |
| 51 | G → T | Leu → Phe | gcgTTGctt | 1 | 1 |  |  |
| 52 | C → T | Leu → Phe | ttgCTTaac |  |  | 1 |  |
| 53 | T → A | Leu → His | ttgCTTaac |  |  | 2 (1) |  |
|  | T → G | Leu → Arg | ttgCTTaac |  |  | 1 |  |
| 56 | A → C | Asn → Thr | cttAACaaa |  | 1 |  |  |
|  | A → T | Asn → Ile | cttAACaaa |  |  | 1 | 1 |
| 57 | C → A | Asn → Lys | cttAACaaa |  |  | 1 |  |
| 58 | A → T | Lys → Stop | aacAAAatc |  |  | 1 |  |
| 59 | A → C | Lys → Thr | aacAAAatc |  |  |  | 1 |
| 64 | G → A | Ala → Thr | atcGCAatg | 4 (1) |  | 1 | 1 |
| 65 | C → T | Ala → Val | atcGCAatg |  |  |  | 1 |
| 68 | T → C | Met → Thr | gcaATGctt |  |  | 1 |  |
|  | T → G | Met → Arg | gcaATGctt | 1 |  |  |  |
| 74 | G → C | Gly → Ala | cttGGAact |  |  |  | 1 |
| 76 | A → G | Thr → Ala | ggaACTgag |  |  | 1 |  |
| 82 | A → G | Lys → Glu | gagAAGaca |  |  | 1 |  |
| 83 | A → T | Lys → Met | gagAAGaca |  |  |  | 2 |
| 88 | G → T | Ala → Ser | acaGCGgaa |  |  |  | 1 |
| 89 | C → G | Ala → Gly | acaGCGgaa |  |  | 1 |  |
|  | C → A | Ala → Glu | acaGCGgaa |  |  | 1 | 1 |
|  | C → T | Ala → Val | acaGCGgaa | 13 (1) | 5 (2) |  |  |
| 94 | G → C | Ala → Pro | gaaGCTgtg |  |  | 1 |  |
|  | G → T | Ala → Ser | gaaGCTgtg |  |  |  | 1 |
| 95 | C → A | Ala → Asp | gaaGCTgtg | 1 |  |  |  |
|  | C → T | Ala → Val | gaaGCTgtg | 1 |  |  |  |
| 97-98 | ­ GT | Frameshift | gctGTGggc | 1 |  |  |  |
| 98 | T → A | Val → Glu | gctGTGggc | 1 |  | 6 (4) | 9 (2) |
|  | T → G | Val → Gly | gctGTGggc |  |  |  | 1 |
| 103 | G → A | Val → Ile | ggcGTTgat | 1 | 1 | 1 | 2 |
|  | G → T | Val → Phe | ggcGTTgat |  |  | 3 |  |
| 104 | T → A | Val → Asp | ggcGTTgat |  |  | 1 |  |
|  | T → C | Val → Ala | ggcGTTgat |  |  |  | 2 (1) |
| 106 | G → T | Asp → Tyr | gttGATaag |  |  | 3 (2) |  |
| 106-108 | + GAT | Frameshift | gttGATaag | 1 |  |  |  |
| 108 | T → A | Asp → Glu | gttGATaag |  |  | 1 | 1 |
| 109 | A → C | Lys → Gln | gatAAGtcg |  |  |  | 1 |
|  | A → G | Lys → Glu | gatAAGtcg |  |  |  | 1 |
|  | A → T | Lys → Stop | gatAAGtcg |  |  | 1 |  |
| 110 | A → C | Lys → Thr | gatAAGtcg |  |  |  | 1 |
|  | A → G | Lys → Arg | gatAAGtcg |  |  |  | 1 |
|  | A → T | Lys → Met | gatAAGtcg |  |  |  | 4 |
| 112 | T → A | Ser → Thr | aagTCGcag |  |  | 1 |  |
| 113 | C → T | Ser → Leu | aagTCGcag |  |  | 1 | 1 |
| 116 | A → T | Gln → Leu | tcgCAGatc |  |  | 6 (2) | 2 |
| 117 | G → T | Gln → His | tcgCAGatc |  |  | 1 |  |
| 118 | A → T | Ile → Phe | cagATCagc | 1 |  |  |  |
| 119 | T → A | Ile → Asn | cagATCagc |  |  |  | 1 |
| 120 | C → G | Ile → Met | cagATCagc |  |  | 1 |  |
| 121 | A → C | Ser → Arg | atcAGCagg |  |  |  | 1 |
| 124 | A → T | Arg → Trp | agcAGGtgg |  |  | 5 (4) | 2 |
| 125 | G → T | Arg → Met | agcAGGtgg |  |  | 1 | 4 (2) |
| 126 | G → T | Arg → Ser | agcAGGtgg |  | 1 | 1 |  |
| 127 | T → A | Trp → Arg | aggTGGaag |  | 1 | 2 | 1 |
| 128 | G → A | Trp → Stop | aggTGGaag |  |  |  | 1 |
|  | G → T | Trp → Leu | aggTGGaag | 1 |  |  |  |
|  | G → C | Trp → cys | aggTGGaag |  |  |  | 1 |
| 129 | G → T | Trp → Cys | aggTGGaag | 1 |  |  |  |
| 131 | A → C | Lys → Thr | tggAAGagg |  |  | 1 |  |
| 132 | G → T | Lys → Asn | tggAAGagg |  |  |  | 1 |
| 133 | A → T | Arg → Trp | aagAGGgac |  |  | 2 | 5 (2) |
| 134 | G → T | Arg → Met | aagAGGgac |  |  | 1 |  |
| 139 | T → A | Trp → Arg | gacTGGatt |  |  | 4 (1) | 2 |
| 141 | G → C | Trp → Cys | gacTGGatt |  |  |  | 1 |
| 142 | A → T | Ile → Phe | tggATTcca | 1 |  |  |  |
| 144 | T → G | Ile → Met | tggATTcca |  |  | 1 |  |
| 146 | C → G | Pro → Arg | attCCAaag |  |  |  | 1 |
| 149 | A → T | Lys → Met | ccaAAGttc |  |  | 15 (2) | 1 |
| 150 | G → T | Lys → Asn | ccaAAGttc | 1 |  |  | 5 (2) |
| 151-152 | ­ T | Frameshift | aagTTCtca |  |  |  | 1 |
| 154 | T → A | Ser → Thr | ttcTCAatg |  |  | 1 | 2 |
| 161 | T → A | Leu → Gln | atgCTGctt |  |  |  | 5 (2) |
|  | T → C | Leu → Pro | atgCTGctt |  |  |  | 3 (1) |
|  | T → G | Leu → Arg | atgCTGctt |  |  | 4 (2) | 1 |
| 164 | T → A | Leu → His | ctgCTTgct |  |  | 2 | 6 (1) |
|  | T → C | Leu → Pro | ctgCTTgct |  |  |  | 2 (1) |
| 165 | T → C | Leu → Leu | ctgCTTgct |  |  | 1 |  |
| 169 | G → T | Val → Phe | gctGTTctt |  |  | 1 |  |
| 172 | C → T | Leu → Phe | gttCTTgaa |  |  | 1 |  |
| 173 | T → A | Leu → His | gttCTTgaa |  |  | 2 |  |
|  | T → C | Leu → Pro | gttCTTgaa |  |  |  | 1 |
| 175 | +GCTT | Frameshift | cttGAAtgg | 1 |  |  |  |
|  | G → T | Glu → Stop | cttGAAtgg |  | 1 | 1 | 1 |
| 176 | A → T | Glu → Val | cttGAAtgg |  |  |  | 1 |
| 177 | A → T | Glu → Asp | cttGAAtgg |  |  |  | 1 |
| 178-185 | + G | Frameshift | gaaTGGGGGGTCgtt | 2 | 6 (3) | 1 | 2 |
| 179 | G → A | Trp → Stop | gaaTGGggg | 1 | 1 |  |  |
| 180 | G → A | Trp → Stop | gaaTGGggg |  |  |  | 1 |
|  | G → T | Trp → Cys | gaaTGGggg |  |  |  | 1 |
| 179-184 | ­ G | Frameshift | gaaTGGGGGGTCgtt |  |  | 1 | 2 |
| 181 | G → T | Gly → Trp | tggGGGgtc |  |  | 1 | 1 |
| 185 | T → C | Val → Ala | gggGTCgtt | 1 |  |  | 1 |
| 187 | G → T | Val → Phe | gtcGTTgac |  |  | 1 |  |
| 191 | A → G | Asp → Gly | gttGACgac |  |  | 1 |  |
|  | A → T | Asp → Val | gttGACgac |  |  | 1 |  |
| 191-192 | AC → TA | Asp → Val | gttGACgac |  |  |  | 2 (1) |
| 192 | C → A | Asp → Glu | gttGACgac |  |  |  | 1 |
| 193 | G → A | Asp → Asn | gacGACgac | 1 |  | 1 | 2 |
|  | G → C | Asp → His | gacGACgac |  |  | 1 |  |
|  | G → T | Asp → Tyr | gacGACgac |  |  | 1 | 1 |
| 196 | G → A | Asp → Asn | gacGACatg | 2 |  | 4 (2) | 2 |
|  | G → C | Asp → His | gacGACatg | 1 |  |  |  |
| 200 | T → A | Met → Lys | gacATGgct |  |  |  | 2 (1) |
| 201 | G → A | Met → Ile | gacATGgct |  | 1 |  |  |
| 202 | G → C | Ala → Pro | atgGCTcga |  |  | 1 |  |
| 206 | G → A | Arg → Gln | gctCGAttg | 1 |  | 1 | 1 |
|  | G → C | Arg → Pro | gctCGAttg |  |  | 2 (1) |  |
| 210 | G → C | Leu → Phe | cgaTTGgcg |  |  |  | 1 |
|  | G → T | Leu → Phe | cgaTTGgcg |  |  |  | 1 |
| 211 | G → A | Ala → Thr | ttgGCGcga |  |  | 1 |  |
| 212 | C → A | Ala → Glu | ttgGCGcga | 2 |  |  | 1 |
|  | C → T | Ala → Val | ttgGCGcga | 2 |  | 2 | 1 |
| 214 | C → T | Arg → STOP | gcgCGAcaa | 1 | 2 (1) |  | 4 |
| 215 | G → C | Arg → Pro | gcgCGAcaa |  |  | 2 | 3 (1) |
| 218 | A → C | Gln → Pro | cgaCAAgtt |  |  | 1 |  |
| 220 | G → C | Val → Leu | caaGTTgct |  |  | 1 |  |
| 223 | G → T | Ala → Ser | gttGCTgcg |  | 1 |  |  |
| 224 | C → A | Ala → Asp | gttGCTgcg | 1 |  |  |  |
| 226 | G → C | Ala → Pro | gctGCGatt |  |  | 1 | 1 |
| 228-229 | GA → TT | Ala Ile → AlaPhe | gctGCGATTctc |  |  |  | 1 |
| 232 | C → A | Leu → Ile | attCTCacc | 1 |  |  |  |
| 241 | A → T | Lys → Stop | aatAAAaaa |  |  |  | 1 |
| 274 | C → A | Gln → Lys | gaaCAAatc |  | 1 |  |  |
| 275 | A → T | Gln → Leu | gaaCAAatc |  |  | 1 |  |
| 289-290 | ­ T | Frameshift | gagTTCtga |  | 1 |  |  |
| 290 | T → A | Phe → Tyr | gagTTCtga |  |  |  | 1 |
| 292 | T → A | Stop → Arg | ttcTGAggt |  | 1 | 2 | 1 |
| 293 | G → T | Stop → Leu | ttcTGAggt |  |  | 1 |  |
|  |  |  |  |  |  |  |  |
| Total |  |  |  | 53 (38) | 33 (25) | 132 (98) | 150 (114) |

^a^ Position 1 is the first base of the start codon in the *cII* coding sequence.

^b^ Presented in term of sequence change on nontranscribed DNA strand.

^c^ Uppercase indicates target codon and target bases are underlined.

Abbreviations: ­, deletion; +, insertion.
